# Compositional Tuning of Barium Titanium Trisulphide‐Based Perovskite Chalcogenides: Manganese and Selenium Substitution Effects on Electronic and Transport Properties

**DOI:** 10.1002/open.70218

**Published:** 2026-05-10

**Authors:** Adel Bandar Alruqi

**Affiliations:** ^1^ Department of Physics Faculty of Science King Abdulaziz University Jeddah Saudi Arabia

**Keywords:** electrical conductivity of chalcogenides, electronic properties of chalcogenides, hall coefficient of chalcogenides, novel materials for photovoltaic applications

## Abstract

The family of transition metal chalcogenides, particularly barium titanium trisulphide ($\text{BaTi}S_{3}$), is one of the classes of materials that have garnered a lot of interest recently, owing to their unique combination of structural versatility, environmental stability, and tunable electronic properties. However, controlled doping of these materials, as is necessary for customizing their electrical and electronic properties, is one of the several challenges still being faced by researchers. Studies on dopant incorporation processes on these materials are yet to be comprehensively determined. In this research paper, two new materials (${\mathrm{BaTi}}_{0.5}{\mathrm{Mn}}_{0.5}S_{3}$ and ${\mathrm{BaTi}}_{0.5}{\mathrm{Mn}}_{0.5}{\mathrm{Se}}_{3}$) were modelled from the $\text{BaTi}S_{3}$ and their electronic and transport properties investigated using density functional theory calculations as implemented in the Quantum Espresso package. The Perdew–Burke–Ernzerhof exchange‐correlation functional was utilized. It was found out that the substitution resulted in lowering the electrical conductivities, carrier mobilities, and formation energies compared to the original sample, while their densities increased. The results also pointed to the adjustment of the materials’ band gaps from 0.71 eV for the original sample to as low as 0.312 eV in the ${\mathrm{BaTi}}_{0.5}{\mathrm{Mn}}_{0.5}{\mathrm{Se}}_{3}$ sample. Regardless of the changes, the Hall coefficients of the samples turned out to be p‐type, whose values increased with the substitutions. The above findings underline the significance of compositional adjustment in maximizing the performance of $\text{BaTi}S_{3}$ and its derivatives in photovoltaic, thermoelectric, and sensor applications.

## Graphical abstract

We investigated the effect of manganese and selenium substitution on BaTiS_3_ using density functional theory calculations performed with Quantum Espresso. Elemental tuning narrows the band gap from 0.71 to 0.312 eV, slightly modifies stability and conductivity, and preserves p‐type behavior, highlighting the potential of controlled doping for energy‐related applications.

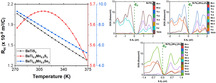

## 1 Introduction

Barium titanium trisulphide ($\text{BaTi}S_{3}$) is one of the chalcogen family of the perovskite structures that have garnered substantial interest, as it shows promising thermoelectric as well as optoelectronic properties. It is structurally comparable to the more studied barium titanate family of oxide‐based materials, but the substitution of oxygen by other chemically close chalcogens such as sulfur, selenium, and tellurium produces significant changes in the electronic as well as structural properties of their materials. $\text{BaTi}S_{3}$ shows interesting quasi‐one‐dimensional (quasi‐1D) structure of the distorted perovskite type. It is normally found to manifest in the crystalline forms of the monoclinic, tetragonal, as well as the orthorhombic structure type. It displays the structure of the chains of TiS_6_ of the metal‐octahedron type, where these chains appear to be connected through corner‐ or edge‐linking along a particular crystallographic type [1, 2].

While the hypothetical perfect arrangement of an ionic perovskite structure follows the ABC arrangement, where titanium occupies the B site and selenium occupying the X site, the real arrangement is a quasi‐1D, as observed in the structure of the particular material (TiS_6_), which possesses a linear chain arrangement in an elongated direction, sharing the faces instead of corners. Such a phenomenon generates a peculiar behavior about the electrical conductivity, as well as the optical and magnetic properties of the material [3, 4, 5].

In essence, $\text{BaTi}S_{3}$ is a semiconductor with a low bandgap and can even fall under the category of low bandgap semiconductors, as its bandgap value is stated to be merely about 0.8 eV [4]. Usually, its bandgap is between 0.5 and 1.25 eV, perhaps due to variations in polymorphism and different synthetic methodologies adopted for its crystallization. First‐principles density functional theory (DFT) calculations have confirmed $\text{BaTi}S_{3}$ to have a relatively direct bandgap and consequently, for energy‐harvesting applications, such materials have a crucial advantage due to efficient directive beam‐absorbing capability [6, 7]. The Fermi level consists explicitly of Ti‐3d and S‐3p states, and have considerable p‐d orbital hybridization, thus making a significant contribution towards enhancing charge transport mechanisms, particularly within the TiS_6_ octahedral chains [8]. Optically, it exhibits a very strong absorption in a wide range of the visible spectrum; the absorption coefficients are higher than 10^5^ cm^−1^, rivaling those of well‐known photovoltaic semiconductors like gallium arsenide and cadmium telluride. Moreover, its optical properties can be tuned systematically through chemical substitution, strain engineering, or dimensionality control, which also involve thin film or nanostructure growth such as nanorods [9].

Various synthesis methods have been developed to prepare $\text{BaTi}S_{3}$, including high‐temperature solid‐state reactions, chemical vapor transport, hydrothermal, and solvothermal syntheses. Early works showed that it can be prepared via controlled reaction of barium sulphide and titanium sulphide in appropriate stoichiometric proportions [10, 11]. Subsequent research has emphasized optimizing phase purity and crystallinity to allow for detailed structural, electronic, and optical characterization. In combination, theoretical methodologies, most notably electronic structure calculations within the DFT framework, have contributed significantly to the understanding of $\text{BaTi}S_{3}$ and its analogues in terms of electronic structure, phase stability, and optical performance. A number of other investigations have explored the consequences of chemical substitution. For example, replacing titanium at the B‐site with transition metals such as iron or cobalt, or substitution of sulfur with heavier chalcogens, notably selenium or tellurium, have been explored as effective ways of tuning the properties of the material [10].

The bandgap of $\text{BaTi}S_{3}$, being optimal for the absorption of light, further increases its possibility to act as one of the best candidates for the absorption of solar energy. As opposed to other methanol‐based perovskite compounds containing halides of lead such as MAPbI_3_, the use of $\text{BaTi}S_{3}$ appears to be beneficial due to the absence of toxic compounds of lead. The ability of the compound to act as one of the candidates for the absorption of light due to its ability to perform well under theoretical calculations and experiments, such as the use of the compound as a thin film, is beneficial for the development of photovoltaics [12, 13].

Apart from its photovoltaic properties, theoretical findings also suggest that $\text{BaTi}S_{3}$ can serve as a material with good mechanical and thermal properties, especially under optimum doped states [14]. In addition, based on its electroactive properties, $\text{BaTi}S_{3}$ also act as an efficient electrocatalytic material, especially towards the electrochemical reaction that occurs during the production of hydrogen gas from a reaction involving carbon dioxide. Indeed, previous findings have shown that $\text{BaTi}S_{3}$ can display efficient electrocatalytic activities, especially upon the incorporation of surface modification techniques/groups with a nanoscale arrangement [9, 11, 15].

Even though considerable achievements have been made in the investigation into the material properties of $\text{BaTi}S_{3}$ as well as related compounds, several challenges are still encountered, particularly with the n‐type or p‐type doping process that can allow the exact tuning of the electrical/electronic properties. Notably, the mechanism concerning the effect of the resultant dopant is still not well understood. For instance, ${\mathrm{BaTi}}_{0.5}{\mathrm{Mn}}_{0.5}S_{3}$ and ${\mathrm{BaTi}}_{0.5}{\mathrm{Mn}}_{0.5}{\mathrm{Se}}_{3}$, which were modeled and then explored in this study for the first time, exemplify how such substitutions can alter formation energies and enhance electrical and electronic properties through the introduction of manganese and selenium.

## 2 Computational Methods

**FIGURE 1:**
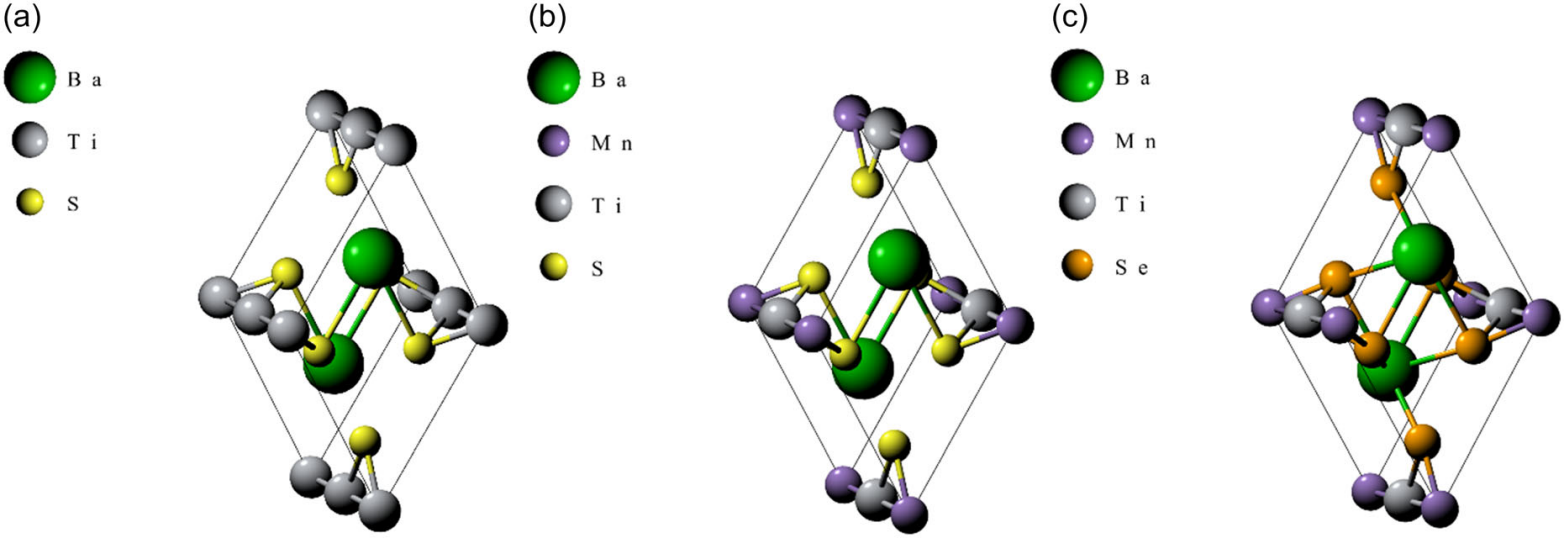
3D structures of the samples: (a) ${\text{BaTiS}}_{3}$, (b) ${\mathrm{BaTi}}_{0.5}{\mathrm{Mn}}_{0.5}S_{3}$, and (c) ${\mathrm{BaTi}}_{0.5}{\mathrm{Mn}}_{0.5}{\mathrm{Se}}_{3}$.

This study employed DFT with *Quantum Espresso* code [16]. The starting material was a hexagonal $\text{BaTi}S_{3}$ cell ($P6_{3}/mmc$ space group), which was downloaded from the Materials Project website (m*p* = 7073) [17]. The original lattice parameters were a=b=6.80 Å and c=5.85 Å (Figure 1a). The cell consisted of 10 atoms: two of barium, two of titanium, and 6 of sulphur. The cell was then modified by replacing one of the two atoms of titanium with manganese to create the ${\mathrm{BaTi}}_{0.5}{\mathrm{Mn}}_{0.5}S_{3}$ cell (Figure 1b), which was further modified by replacing all the sulphur atoms with those of selenium to create the ${\mathrm{BaTi}}_{0.5}{\mathrm{Mn}}_{0.5}{\mathrm{Se}}_{3}$ cell (Figure 1c). The modelling of the structures was done using *Burai* [18], a graphical user interface for *Quantum Espresso*.

All the samples were then optimized in terms of: kinetic energy cut‐off (ecut), charge density cut‐off (ecutrho), K_points, lattice parameters, and lattice points. The calculation made use of the Perdew–Burke–Ernzerhof exchange‐correlation functional, with projector augmented wave pseudopotentials: *Ba.pbe‐spn‐kjpaw_psl.1.0.0.UPF*, *Ti.pbe‐spn‐kjpaw_psl.1.0.0.UPF*, *S.pbe‐n‐kjpaw_psl.1.0.0.UPF*, *Se.pbe‐dn‐kjpaw_psl.1.0.0.UPF*, and *Mn.pbe‐spn‐kjpaw_psl.0.3.1.UPF*. The ecut values were varied from 10 to 90 Ry in steps of 10 Ry for all the cells, K_points from 2 2 2 to 9 9 9 in steps of 2 2 2, parameter a from 6.12 to 7.48 Å (10% below and above the reference value) in steps of 0.113 Å (13 data points), and parameter c from 5.265 to 6.435 Å (10% below and above the reference value) in steps of 0.0975 Å (13 data points). The lattice points were optimized through variable‐cell relaxation using the Broyden–Fletcher–Goldfarb–Shanno algorithm [19]. To check the structural stabilities of the samples, their formation energies as well as tolerance factors were computed. The formation energies were calculated according to the method by Alruqi et al. [20]:

(1) $$H_{f}\left(Q\right)=E_{\mathrm{tot}}\left(Q\right)-\sum_{i}n_{i}{\mu_{\mathrm{chem}}}_{i}$$

where $Q=\text{BaTi}S_{3}$, ${\mathrm{BaTi}}_{0.5}{\mathrm{Mn}}_{0.5}S_{3}$, $\mathrm{or}\;{\mathrm{BaTi}}_{0.5}{\mathrm{Mn}}_{0.5}{\mathrm{Se}}_{3}$; *n* is the number of atoms of each atomic species in each compound; and ${\text{µ}}_{\text{chem}}$ is the chemical potential (the total energy per atom of element *i*).

The Goldschmidt tolerance analysis was performed according to Szeleszczuk et al. [21]:

(2) $$t=\frac{r_{A}+r_{X}}{\sqrt{2}\left(r_{B}+r\_X\right)}$$

where $r_{A}$ is the radius of the A‐site (barium), $r_{B}$ is the radius of the B‐site (titanium or manganese), and $r_{X}$ is the radius of the anion (sulphur or selenium).

The computation of the electrical properties was done using the *BolzTrap2* code, which is widely used for calculating semiclassical transport coefficients based on electronic band structure data. It calculates electronic transport properties using the semiclassical Boltzmann transport equation in the constant relaxation time approximation [22]

(3) $${\text{σ}}_{\text{αβ}}\left(\text{µ},T\right)=e^{2}\int d\text{ε}\left(-\frac{\partial f\left(\text{εµ},T\right)}{d\text{ε}}\right)\sum_{\text{αβ}}\left(\text{ε}\right)$$

where σ is the electrical conductivity, *α, β* are the Cartesian components (*x*, *y*, *z*); f(εµ,T) is the Fermi–Dirac distribution; and $\sum_{\text{αβ}}(\text{ε})$ is the transport distribution function. The carrier concentration (n) and the carrier mobility (${\text{µ}}_{\mathrm{mob}}$) were calculated according to the work by Wang et al. [23]:

(4) $$n=\frac{N}{V_{\mathrm{cell}}}$$

(5) $$\mu_{\mathrm{mob}}=\frac{\sigma }{ne}$$

where *N* is the number of electrons/holes in the unit cell, $V_{\text{cell}}$ is the unit cell volume, and *e* is the charge of electron.

The Hall coefficient tensor $\left(R_{\mathrm{ijk}}\right)$ on the other hand, is derived from the third‐order conductivity tensor [24]:

(6) $$R_{\mathrm{ijk}}=\frac{1}{{\text{σ}}_{\mathrm{ii}}{\text{σ}}_{\mathrm{jj}}}{\text{ε}}_{\mathrm{klm}}{\text{σ}}_{\text{ijlm}}^{\left(2\right)}$$

The Hall coefficient $\left(R_{H}\right)$ is then given by:

(7) $$R_{H}=\frac{1}{{\text{σ}}^{2}}{\text{σ}}^{\left(2\right)}=\frac{1}{ne}$$

All the transport properties were calculated within a temperature range of 273–373 K. The electronic properties were calculated by first performing self‐consistent function (SCF) calculations on the relaxed structures using a 5 5 6 K_point mesh, followed by nonself‐consistent function (NSCF) calculations using a denser 15 15 18 K_point mesh. Bands calculations were then performed using the HSE06 hybrid functional by setting the following parameters in both the SCF and NSCF input files: *input_dft = ‘hse’*; *exx_fraction = 0.25*; and *screening_parameter = 0.106*. The following high‐symmetry path was used: Γ−M−K−Γ−A−L−H−A−LM−H−K. The band structure and density of states (DOS) data were then extracted for plotting.

## 3 Results and Discussion

### 3.1 Structural Properties

**FIGURE 2:**
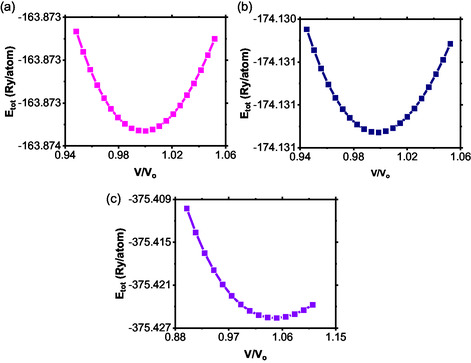
The variation of total energy with normalized unit cell volume for: (a) $\text{BaTi}S_{3}$, (b) ${\mathrm{BaTi}}_{0.5}{\mathrm{Mn}}_{0.5}S_{3}$, and (c) ${\mathrm{BaTi}}_{0.5}{\mathrm{Mn}}_{0.5}{\mathrm{Se}}_{3}$.

The structural properties of a material have a strong impact when responding to outside influences such as stress, temperature changes, and exposure to the environment. It is clearly evident from Figure 2 that all the samples depict smooth parabolic energy‐volume curves, showing good stability in the structures. Moreover, the distinct minima imply their good crystal structure and low compressibility. As to the ${\mathrm{BaTi}}_{0.5}{\mathrm{Mn}}_{0.5}S_{3}$ structure (Figure 2b), the curve of the total energy has a more extended minimum, which can be due to its increased compressibility. The reasons behind this could lie in the distortions of the lattice originating from the substitution of the titanium ion by the manganese ion due to their different ionic radii. While ${\mathrm{BaTi}}_{0.5}{\mathrm{Mn}}_{0.5}S_{3}$ remains stable, it may be more responsive to external pressures or strains, a property that can be advantageous in applications such as piezoelectric devices or pressure sensors. In the case of ${\mathrm{BaTi}}_{0.5}{\mathrm{Mn}}_{0.5}{\mathrm{Se}}_{3}$ (Figure 2c), the energy curve is also smooth, but shows a higher energy value, indicating that its configuration is less stable compared to the other two materials.

$\text{BaTi}S_{3}$ underwent a contraction upon relaxation in both a and c axes as shown in Table 1, since the values are slightly lower than those of the reference values [17]. However, they are quite in accord with the previous computational work by Paudel & Tsymbal [7], and experimental work by Fu et al. [5]. A slight reduction in both a and c axes observed in ${\mathrm{BaTi}}_{0.5}{\mathrm{Mn}}_{0.5}S_{3}$ is consistent with the smaller ionic radii of Mn^4+^ (0.8 Å) against Ti^4+^ (1.0 Å). However, selenium substitution was observed to increase parameter a, which is consistent with the larger Se^2‐^ ionic size (at 1.98 Å) compared to that of S^2‐^ (at 1.84 Å), leading to expansion of the lattice.

**TABLE 1:** The computed structural properties of the materials.

Material	*a*, Å	*c*, Å	*c*/*a*	*ρ* $,\;\mathrm{kg}/m^{3}$	$E_{f},\;\text{eV}/\text{atom}$	*t*
$\text{BaTi}S_{3}$	6.798, 6.73 [7], 6.765 [5]	5.835 5.92 [7], 5.793 [5]	0.858	4,002, 3,980 [25]	−5.54	0.84
${\mathrm{BaTi}}_{0.5}{\mathrm{Mn}}_{0.5}S_{3}$	6.747	5.644	0.837	4,253	−5.40	0.86
${\mathrm{BaTi}}_{0.5}{\mathrm{Mn}}_{0.5}{\mathrm{Se}}_{3}$	6.895	5.836	0.846	5,884	−4.86	0.99

The calculated densities of the materials increased with the substitution of manganese and selenium, most likely due to the heavier masses of these elements. However, these densities fall within a range similar to that of well‐known thermoelectric materials, such as PbTe (8,100–8,200 kg/m^3^) [26] or Bi_2_Te_3_ (7,642 kg/m^3^) [27]. This indicates that the materials are of moderate density and thus, can be considered for the formation of thin film materials. Formation energy is usually considered an essential aspect of the thermodynamic stability of materials in the context of compound formation. A more negative formation energy indicates higher stability of a compound. All the materials considered in this study were found to possess negative formation energy; hence, all are considered stable in the thermodynamic context. From the various materials considered in this work, the pristine $\text{BaTi}S_{3}$ was found to have the highest stability. However, partial replacement of the titanium site in the compound ${\mathrm{BaTi}}_{0.5}{\mathrm{Mn}}_{0.5}S_{3}$ with manganese atom causes instability in the compound. However, this compound still possesses relatively favorable energy as indicated in Table 1. Replacement at the sulfur site in ${\mathrm{BaTi}}_{0.5}{\mathrm{Mn}}_{0.5}{\mathrm{Se}}_{3}$ with selenium atom causes instability in the compound due to the large atomic radius of selenium with relatively inferior bonding interactions.

The Goldschmidt tolerance factor provides a clear framework for understanding the structural trends across the investigated structures. $\text{BaTi}S_{3}$ exhibits a moderate tolerance factor (Table 1), indicating a significant deviation from the ideal cubic perovskite structure. As a result, it adopts a quasi‐1D structure composed of face‐sharing TiS_6_ octahedral chains. Although there is no tolerance factor specifically for $\text{BaTi}S_{3}$ in the literature, the tolerance factors for perovskites usually range between 0.8 and 1.0 [28]. Upon partial substitution of Ti^4+^ with Mn^4+^ in ${\mathrm{BaTi}}_{0.5}{\mathrm{Mn}}_{0.5}S_{3}$, the tolerance factor increases slightly, due to the smaller ionic radius of Mn^4+^. This leads to a modest reduction in the lattice distortion, although the structure remains noncubic and retains low‐dimensional properties. A more pronounced structural transition occurs in ${\mathrm{BaTi}}_{0.5}{\mathrm{Mn}}_{0.5}{\mathrm{Se}}_{3}$, where the substitution of S^2−^ with the larger Se^2−^ anion increases the tolerance factor to almost 1, indicating a strong tendency towards a 3D cubic or near‐cubic perovskite structure with corner‐sharing octahedra [29].

### 3.2 Electrical Properties

**FIGURE 3:**
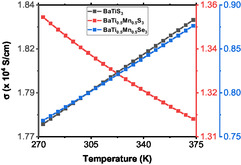
The variation of electrical conductivities of the three materials with temperature.

Figure 3 demonstrates how temperature affects the electrical conductivity for all the materials across the entire temperature range, which encompasses material device usage close to and above the normal human body operating temperatures. For example, ${\text{BaTiS}}_{3}$ demonstrates moderate levels of electrical conductivity that increase with increase in temperature, akin to a semiconducting material. Such a behavior is desired, as it demonstrates thermally activated conduction, as thermal energy allows charge carriers to overcome band gaps, making it an appropriate material for solar cells and sensors [27]. ${\mathrm{BaTi}}_{0.5}{\mathrm{Mn}}_{0.5}S_{3}$ demonstrates a low level of electrical conductivity as opposed to ${\text{BaTiS}}_{3}$ across the temperature range as demonstrated in Figure 3 and Table 2. This suggests that the substitution of manganese introduces local states and scattering centers, which reduces the mobility of charge carriers.

**TABLE 2:** The computed electrical and electronic properties of the three materials.

Material	$\text{BaTi}S_{3}$	${\mathrm{BaTi}}_{0.5}{\mathrm{Mn}}_{0.5}S_{3}$	${\mathrm{BaTi}}_{0.5}{\mathrm{Mn}}_{0.5}{\mathrm{Se}}_{3}$
$\text{RH}(\times 10^{-9}\;m^{3}/C)$	1.849	5.645	8.276
$\sigma (\times 10^{4}S/\mathrm{cm})$	1.789	1.339	0.794
${µ}_{\mathrm{mob}}$ (cm^2^/V•s)	301, 300 [3]	225	133
$E_{g}\;(\mathrm{eV})$	0.71, 0.8 [4], 0.5 [7]	0.69	0.312

When comparing the materials to standard doped silicon, which has an electrical conductivity of 4.37 × 10^4^ S/cm at room temperature [30], both $\text{BaTi}S_{3}$ and ${\mathrm{BaTi}}_{0.5}{\mathrm{Mn}}_{0.5}S_{3}$ perform comparably. However, ${\mathrm{BaTi}}_{0.5}{\mathrm{Mn}}_{0.5}S_{3}$ exhibits a unique behavior: its electrical conductivity decreases as the temperature rises. This is unusual for semiconductors, and can occur in systems that are moderately to heavily doped, where charge carriers move through hopping conduction in disordered materials. In doped semiconductors, the dopants become fully ionized at a certain temperature, after which the concentration of charge carriers stabilizes. Among all the materials studied in this work, ${\mathrm{BaTi}}_{0.5}{\mathrm{Mn}}_{0.5}{\mathrm{Se}}_{3}$ has the lowest electrical conductivity, as shown in Table 2, but it displays a temperature trend similar to that of $\text{BaTi}S_{3}$. This lower electrical conductivity may be due to an increased effective mass or stronger interactions between electrons and phonons because of the presence of selenium. It is important to note that, to the best of our knowledge, no experimental literature values are available for the electrical conductivity of $\text{BaTi}S_{3}$, thus limiting direct comparison. Although a high electrical conductivity is often advantageous for many electronic applications, a lower conductivity can be favorable in thermoelectric materials. In such systems, reduced electrical and thermal conductivities can contribute to improved performance metrics, including an enhanced thermoelectric figure of merit [31].

**FIGURE 4:**
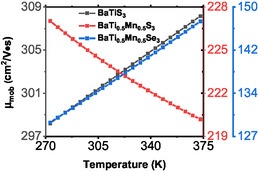
The computed carrier mobility against temperature for all the samples.

Figure 4 presents the calculated carrier mobility as a function of temperature for $\text{BaTi}S_{3}$ and its substituted counterparts in the temperature range of 270–375 K. At 300 K, the carrier mobility of $\text{BaTi}S_{3}$ is in excellent agreement with some reported literature values as shown in Table 2 [4]. This agreement validates the reliability of the computational approach and the assumptions employed in the transport calculations in this study. Furthermore, the mobility of $\text{BaTi}S_{3}$ exhibits a slight increase with temperature, suggesting that band‐like transport dominates and that impurity scattering plays a relatively minor role in this system.

In contrast, partial substitution of titanium with manganese leads to a noticeable reduction in the carrier mobility. For ${\mathrm{BaTi}}_{0.5}{\mathrm{Mn}}_{0.5}S_{3}$, this represents a reduction of about 33% compared to the pristine compound (Table 2). The decrease can be attributed to enhanced scattering arising from the Mn‐induced disorder and possible localized electronic states, which impede charge carrier transport. Additionally, the mobility of this composition decreases with increasing temperature, indicating that phonon scattering becomes the dominant limiting mechanism. A more pronounced reduction in the mobility is observed for ${\mathrm{BaTi}}_{0.5}{\mathrm{Mn}}_{0.5}S_{3}$, where the mobility at 300 K is 126% lower than that of the pristine sample. The substitution of sulfur with the heavier selenium atom likely introduced stronger lattice vibrations, leading to increased electron–phonon scattering and consequently, a lower carrier mobility [32]. Despite this reduction, the carrier mobility of ${\mathrm{BaTi}}_{0.5}{\mathrm{Mn}}_{0.5}{\mathrm{Se}}_{3}$ shows an increasing trend with temperature, suggesting a complex interplay between scattering mechanisms and band structure effects.

**FIGURE 5:**
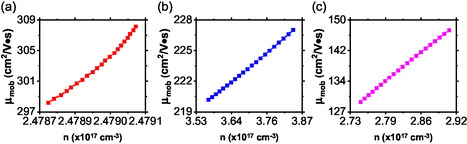
The calculated carrier mobility against carrier concentration for (a) $\text{BaTi}S_{3}$, (b) ${\mathrm{BaTi}}_{0.5}{\mathrm{Mn}}_{0.5}S_{3}$, and (c) ${\mathrm{BaTi}}_{0.5}{\mathrm{Mn}}_{0.5}{\mathrm{Se}}_{3}$.

Figure 5 illustrates the variation of carrier mobility as a function of carrier concentration for all the samples. The carrier concentration of all systems lies in the order of 10^17^ cm^−3^, which is consistent with the reported values for related chalcogenide perovskites such as BaZrS_3_ [33]. This agreement indicates that the studied materials fall within a realistic doping regime relevant for semiconductor and thermoelectric applications. For BaTiS_3_ (Figure 5a), the carrier mobility increases monotonically with carrier concentration within the studied concentration window. This behavior suggests that the increase in carrier concentration enhances electrical transport, likely due to improved carrier screening and reduced relative impact of scattering centers. The high mobility values observed in this regime further confirm the good transport properties of BaTiS_3_. A similar increasing trend is observed for ${\mathrm{BaTi}}_{0.5}{\mathrm{Mn}}_{0.5}S_{3}$ (Figure 5b), although with reduced mobility values. The lower mobility of ${\mathrm{BaTi}}_{0.5}{\mathrm{Mn}}_{0.5}S_{3}$ compared to that of BaTiS_3_ reflects the influence of manganese substitution, which introduces disorder and enhances carrier scattering. Nevertheless, the positive correlation between mobility and carrier concentration indicates that increased doping partially compensates for scattering effects by facilitating carrier transport [34]. In the case of ${\mathrm{BaTi}}_{0.5}{\mathrm{Mn}}_{0.5}{\mathrm{Se}}_{3}$ (Figure 5c), the mobility also exhibits a monotonic increase with carrier concentration. The overall reduction in the mobility relative to the sulfide counterparts can be attributed to the heavier selenium atoms, which enhance lattice vibrations and electron–phonon interactions. Despite this, the consistent upward trend with increasing carrier concentration suggests that transport remains governed by band‐like conduction mechanisms.

**FIGURE 6:**
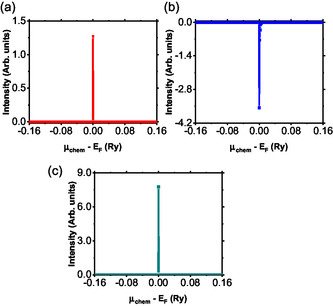
The Hall coefficient against energy for (a) $\text{BaTi}S_{3}$, (b) ${\mathrm{BaTi}}_{0.5}{\mathrm{Mn}}_{0.5}S_{3}$, and (c) ${\mathrm{BaTi}}_{0.5}{\mathrm{Mn}}_{0.5}{\mathrm{Se}}_{3}$.

Figure 6 presents the energy dependence of the Hall coefficient for all the investigated materials. The Hall coefficient is a key transport parameter that provides insight into the dominant charge carriers, their mobility, and electronic band structure properties, particularly in the vicinity of the Fermi level. For both $\text{BaTi}S_{3}$ and ${\mathrm{BaTi}}_{0.5}{\mathrm{Mn}}_{0.5}{\mathrm{Se}}_{3}$, the Hall coefficients remain positive near the Fermi level, indicating that hole carriers dominate charge transport (Figure 6a,c). These positive values are characteristic of p‐type conductivity and are consistent with a quasi‐1D electronic structure along the Ti–S chains, as well as the presence of a relatively small band gap in $\text{BaTi}S_{3}$ [4, 7]. The relatively stable Hall coefficient near the Fermi level suggests a moderate effective mass for the charge carriers and only minor variations in the DOS in this energy region, which is consistent with the semiconducting nature of the material [35]. Moreover, ${\mathrm{BaTi}}_{0.5}{\mathrm{Mn}}_{0.5}{\mathrm{Se}}_{3}$ exhibits attractive properties for use in thermoelectrics, high‐sensitivity Hall sensors, and adaptive electronic devices, which are the areas where material responses to small energy perturbations are highly regarded.

The profile of the Hall coefficient of ${\mathrm{BaTi}}_{0.5}{\mathrm{Mn}}_{0.5}S_{3}$, as shown in Figure 6b, reveals a significant energy dependence. In such cases, the presence of the negative minimum energy below the Fermi level with a rapid increase to positive values above the Fermi level reveals implications of the complex nature of the multiband electronic structure of the material. The presence of the negative value of the Hall coefficient near the Fermi level may reveal increased dominance of the properties of the n‐type conduction, which may arise due to the presence of manganese incorporated in material [36]. Therefore, based on the results of this study, the properties of the electronic band structure of the n‐type modelled compound may reveal the implications of the possibility of tailoring its electronic properties according to the development of the synthesis protocol. The implications of these results reveal the ability to create the conditions for the ambipolar properties of the Hall coefficient of the compound, where the n‐ and p‐conducting properties may arise within the compound according to the specific synthesis protocols developed to create the desired spin‐field effect transistors [37].

**FIGURE 7:**
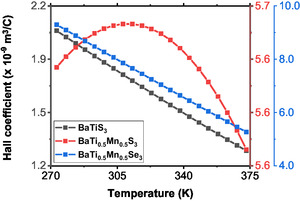
The computed Hall coefficients of the three samples as functions of temperature.

As presented in Figure 7, the Hall coefficient of $\text{BaTi}S_{3}$ retains its positive and consistent value across the entire temperature range. It is crucial to note that the Hall coefficient always increases linearly when a material is a p‐type semiconductor. Additionally, the slow rate at which the Hall coefficient increases with temperature reveals a nearly constant concentration for a semiconductor, which is a property often displayed by materials considered to have low levels of doping. It also reveals low values for the concentration of holes when the temperature increases, which is consistent with low values for a band gap [4, 7].

In ${\mathrm{BaTi}}_{0.5}{\mathrm{Mn}}_{0.5}S_{3}$, the Hall coefficient increases with temperature to a maximum value at about 308 K and then decreases slowly, thus taking on a parabolic temperature dependence. The increase in the Hall coefficient in the lower temperature range is indicative of p‐type conduction. However, in contrast to $\text{BaTi}S_{3}$, this compound shows stronger sensitivity to temperature, which may be believed to indicate a higher hole concentration due to thermal activation process. The substitution of manganese for the titanium site is likely to introduce acceptor‐like states in $\text{BaTi}S_{3}$, and accounts for better hole generation at higher temperatures. This behavior reflects multiple types of charge carriers. Additionally, the change in the slope could also be related to higher scattering or minor electron‐like contribution at low temperatures.

${\mathrm{BaTi}}_{0.5}{\mathrm{Mn}}_{0.5}{\mathrm{Se}}_{3}$ exhibits the most pronounced decrease in the Hall coefficient with temperature, as shown in Figure 7, indicating a dominant thermally activated p‐type conduction. Replacing sulphur with selenium, which has a larger ionic radius due to enhanced polarizability, may change the electronic configuration or decrease the activation energy for hole transport by decreasing the band gap. The rapid variation of the Hall coefficient with temperature indicates that the carrier concentration, which would be small at lower temperatures, rises appreciably with increasing temperature, as expected in materials having a small gap between bands in the band structure of a semiconductor. To put this into perspective, as presented in Table 2, the Hall coefficients of the two novel compounds have increased compared to the pristine compound. This can be attributed to increased thermally activated generation of charge carriers. The compound ${\mathrm{BaTi}}_{0.5}{\mathrm{Mn}}_{0.5}{\mathrm{Se}}_{3}$ shows substantial variations in Hall coefficients at different temperatures. Therefore, it shows promise to be used in applications of high dependence on temperature‐related changes in electric properties, such as sensors, and also as thermoelectric materials [37]. Also, from the analysis of the results presented in Table 2 and Figure 7, the Hall coefficients of the modeled compounds have increased compared to the pristine $\text{BaTi}S_{3}$, showing low‐concentration of charge carriers. It should be noted that there is lack of experimental Hall coefficients of $\text{BaTi}S_{3}$ in the literature.

### 3.3 Electronic Properties

**FIGURE 8:**
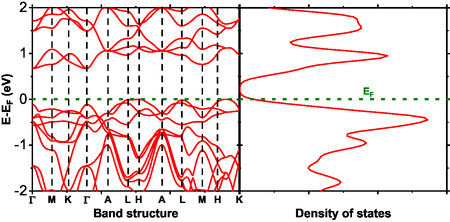
Band structure and total DOS of $\text{BaTi}S_{3}$ as a function of energy.

**FIGURE 9:**
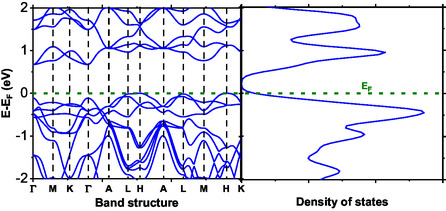
Band structure and DOS of ${\mathrm{BaTi}}_{0.5}{\mathrm{Mn}}_{0.5}S_{3}$ as a function of energy.

**FIGURE 10:**
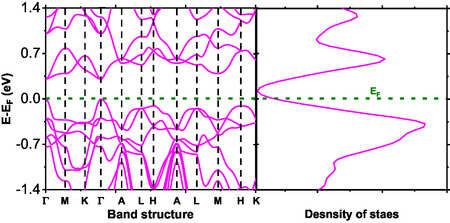
Band structure and DOS of ${\mathrm{BaTi}}_{0.5}{\mathrm{Mn}}_{0.5}{\mathrm{Se}}_{3}$ as a function of energy.

The knowledge of the electronic structure is imperative in predicting the physical properties of a particular compound and evaluating its applicability in electronics and photonics. The electron energy level plots in a crystal momentum index in the Brillouin zone are clearly depicted in the band structure in Figures 8–10. The compounds studied in this work display an indirect band gap. In Figure 8, $\text{BaTi}S_{3}$ displays an energy gap of a few electron volts. It also displays a minimum energy level in the valance and conduction bands, corresponding to other k‐points. However, the results are in direct agreement with previous findings on compounds having an energy gap of a few eV. The compounds display excellent applicability in photovoltaics and field effect transistor devices, since they clearly display nonzero energy gaps, thus qualifying as one of the requirements of a semiconductor.

As depicted in Figure 9, ${\mathrm{BaTi}}_{0.5}{\mathrm{Mn}}_{0.5}S_{3}$ presents a smaller band gap compared with the pristine $\text{BaTi}S_{3}$, suggesting a tendency towards narrow‐gap semiconducting or weakly metallic behavior. Despite this reduction, the band gap remains indirect and is along the Γ‐L high‐symmetry point. The introduction of manganese significantly changes the electronic structure, since Mn‐d orbitals add extra states around the Fermi level. The DOS analysis shows that these d‐derived states can either reduce the band gap or introduce mid‐gap states, hence influencing the material's electronic and transport properties. Additionally, the presence of Mn‐d orbitals may give rise to localized magnetic moments and thus, point to magnetic ordering or spin polarization. These properties emphasize its prospect for application in spintronic devices, which require coupled electronic and magnetic properties [38].

The band structure of ${\mathrm{BaTi}}_{0.5}{\mathrm{Mn}}_{0.5}{\mathrm{Se}}_{3}$, as depicted in Figure 10, bears some similarities to that of $\text{BaTi}S_{3}$ and ${\mathrm{BaTi}}_{0.5}{\mathrm{Mn}}_{0.5}S_{3}$; nonetheless, it displays an appreciably reduced band gap. This significant reduction can mainly be ascribed to changes in the structure, which are brought about by replacing sulfur with selenium. Compared to sulfur, selenium displays a larger atomic radius and a relatively low electronegativity. As a result, it provides p‐states of appreciably low energy and consequently, affects the valence band, eventually leading to the emergence of a narrower band gap as depicted by its band structure and DOS. Moreover, it can be established that ${\mathrm{BaTi}}_{0.5}{\mathrm{Mn}}_{0.5}{\mathrm{Se}}_{3}$ seems to display metallic or semimetallic properties based on their increased DOS at the Fermi surface.

**FIGURE 11:**
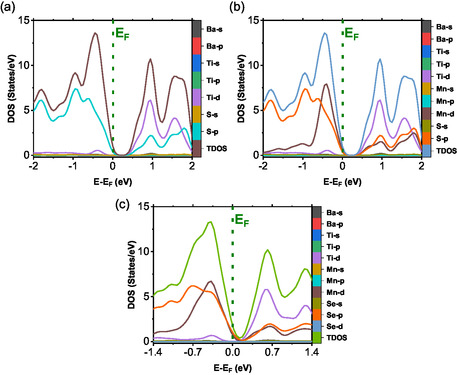
The calculated DOS of (a) $\text{BaTi}S_{3}$, (b) ${\mathrm{BaTi}}_{0.5}{\mathrm{Mn}}_{0.5}S_{3}$, and (c) ${\mathrm{BaTi}}_{0.5}{\mathrm{Mn}}_{0.5}{\mathrm{Se}}_{3}$ as functions of energy.

The DOS analysis shown in Figure 11a indicates that the valence band edge of $\text{BaTi}S_{3}$ is dominated by S‐p and Ti‐d states, whereas the conduction band is primarily composed of Ti‐d states. Contributions from Ba‐s and Ba‐p orbitals near the Fermi level are negligible. This electronic distribution is characteristic of perovskite chalcogenides, in which transport properties are largely governed by transition‐metal d states, while the chalcogen p states define the valence band. Upon manganese substitution, as illustrated in Figure 11b, the Mn‐d orbitals make substantial contributions to both the valence and conduction bands. These Mn‐d states overlap with Ti‐d states, enabling hybridization that may give rise to magnetic ordering or a half‐metallic behavior.

From Figure 11c, the Se‐p states are more delocalized compared to the S‐p states (Figure 11a). This implies a variation in the DOS. This is similar to the behavior observed in the case of ${\mathrm{BaTi}}_{0.5}{\mathrm{Mn}}_{0.5}S_{3}$, as depicted in Figure 11b, where the Mn‐d orbitals are dominant. This behavior guarantees a high DOS at the Fermi level, which could improve the carrier concentration. This, in turn, would enhance the performance of thermoelectric properties [39]. Band gap tuning using the combined substitution of manganese and selenium can improve the range of the solar absorption spectrum. This, consequently, enhances the possible efficiency of tandem solar cells. The incorporation of manganese is additionally suggested through the provision of unpaired electrons, which could improve magnetic order. Even in the presence of spin polarization at the Fermi level, the above‐determined compounds, that is, ${\mathrm{BaTi}}_{0.5}{\mathrm{Mn}}_{0.5}S_{3}$ and ${\mathrm{BaTi}}_{0.5}{\mathrm{Mn}}_{0.5}{\mathrm{Se}}_{3}$, could act as magnetic semiconductors or spin injectors.

## 4 Conclusion

The results of this work illustrate the potential of $\text{BaTi}S_{3}$‐based compounds for various advanced optoelectronic and thermoelectric applications, while demonstrating a path to systematically tune their physical properties through targeted elemental substitution. From a structural aspect, all the compounds are thermodynamically stable, as reflected from their negative formation energies with smooth and well‐defined energy–volume curves. Electrical conductivity analysis reveals that $\text{BaTi}S_{3}$ behaves as a typical semiconductor with conductivity increasing with temperature; whereas after the partial substitution of titanium by manganese, the electrical conductivity reduced with increase in temperature, suggesting enhanced carrier scattering, possibly arising either from local lattice disorder or dopant‐induced electronic states. Remarkably, the temperature‐dependent electrical conductivity of ${\mathrm{BaTi}}_{0.5}{\mathrm{Mn}}_{0.5}S_{3}$ mimics that of disordered or heavily doped semiconductors, reflecting a possible tunability of conduction mechanisms via controlled doping. ${\mathrm{BaTi}}_{0.5}{\mathrm{Mn}}_{0.5}{\mathrm{Se}}_{3}$, on the other hand, presents the lowest electrical conductivity, which is in agreement with stronger electron–phonon interactions and a larger effective charge carrier mass. The carrier mobility decreased with increasing compositional disorder and atomic mass, whereas all the materials exhibited a similar dependence of carrier mobility against carrier concentration, with mobility increasing as the carrier density increased within the studied temperature range. The Hall coefficient increased with increase in temperature, with ${\mathrm{BaTi}}_{0.5}{\mathrm{Mn}}_{0.5}{\mathrm{Se}}_{3}$ having the highest value at 8.276 $\times \;10^{-9}\;m^{3}/C$. Band‐gap calculations confirmed that $\text{BaTi}S_{3}$ is a narrow‐band‐gap semiconductor with a calculated band gap of 0.71 eV, which is comparable to the well‐established photovoltaic absorbers. There was a minor decrease in the manganese‐based compounds and a large reduction in the selenium‐based compound, resulting in a reduced band gap to 0.690 eV and 0.312 eV for ${\mathrm{BaTi}}_{0.5}{\mathrm{Mn}}_{0.5}S_{3}$ and ${\mathrm{BaTi}}_{0.5}{\mathrm{Mn}}_{0.5}{\mathrm{Se}}_{3}$ respectively. The reduction of the energy gap in response to the manganese and selenium substitutions could be beneficial in light of improved near infrared‐bandgap absorptions. However, they could negatively affect heat dissipation in solar panel devices. The consequence of altering the energy gap is obvious and desirable in the synthesis of compounds tailored for certain solar panel designs. The compounds discussed in this work all exhibit positive Hall coefficients, thus showing dominant p‐type properties. The addition of manganese and selenium has increased the magnitude of the Hall coefficient. The p‐type nature of the materials is of vast use, especially when complemented by compounds of n‐type properties.

## Funding

This study was supported by the Deanship of Scientific Research (DSR) at King Abdulaziz University, Jeddah, Saudi Arabia, under grant no. (IPP:1096‐130‐2025).

## Conflicts of Interest

The author declares no conflicts of interest.

## Data Availability

The data that support the findings of this study are available from the corresponding author upon reasonable request.
